# ASO Author Reflections: The Role of Beta-Blockers in Gastric Cancer—A Pathway to Personalized Therapy?

**DOI:** 10.1245/s10434-025-17326-5

**Published:** 2025-04-24

**Authors:** Nerma Crnovrsanin, Sarah Zumsande, Ingmar F. Rompen, Sabine Schiefer, Sarah Zimmer, Wenjun Hu, Johanna Arnscheidt, Fritz Brinkmann, Thomas Longerich, Georg Martin Haag, Thomas Schmidt, Mohammed Al-Saeedi, Leila Sisic, Henrik Nienhüser

**Affiliations:** 1 Department of General, Abdominal and Transplantation Surgery, Heidelberg University Hospital, Heidelberg, Germany; 2 Department of Pathology, Heidelberg University Hospital, Heidelberg, Germany; 3 Department of Medical Oncology, National Center of Tumor Diseases, Heidelberg University Hospitam, Heidelberg, Germany; 4 Department of General, Abdominal, Tumor and Transplantation Surgery, Cologne University Hospital, Cologne, Germany

## Past

Chronic stress, via local or systemic inflammation, fosters cancer development by altering immune responses, aggravating key tumor hallmarks, and engaging adrenergic, cholinergic, and neurotrophin signaling, establishing the nervous system as a key tumor microenvironment component.^1^ Preclinical studies have demonstrated that beta-adrenergic receptor (ADRB) activation promotes tumor growth, resistance to chemotherapy, and increased angiogenesis. Beta-blockers (BB), commonly used for cardiovascular diseases, have been suggested to counteract these effects and improve cancer outcomes.^2^ Whereas retrospective studies and meta-analyses have shown potential survival benefits and better treatment responses in cancers, such as breast and prostate, evidence in gastric cancer (GC) has been scarce and conflicting.

## Present

Our study, the largest retrospective cohort to assess BB use in European GC patients to date, demonstrated that BB intake had no significant effect on overall survival, recurrence-free survival, or response to neoadjuvant chemotherapy in the overall cohort of locally advanced GC patients.^3^ However, when stratified by histopathological subtype, a striking difference emerged: BB use was independently associated with improved overall survival and recurrence-free survival in patients with diffuse-type gastric cancer (DGC), whereas a negative trend was observed in intestinal-type gastric cancer. Furthermore, our immunohistochemical analysis revealed that BB users exhibited higher ADRB2 expression, suggesting a possible compensatory upregulation. Notably, in DGC, increased ADRB2 expression was associated with lower nodal burden but higher local tumor innervation. A key limitation of our study is that we did not have information on the microsatellite instability subtype in our cohort, which could have influenced the results, particularly given the established prognostic and predictive role of microsatellite instability in GC.

## Future

The findings of our study underscore the importance of considering GC histopathological subtypes when evaluating the potential benefits of BB therapy. Future research should focus on prospective trials to validate the impact of BB on GC outcomes, particularly in DGC patients. An ongoing clinical trial (NCT05651594) is currently investigating the addition of BB to pembrolizumab-based immunotherapy in patients with unresectable GC. Further interventional studies are needed to assess the role of BB in localized GC, particularly in combination with standard treatments and to better understand its potential synergy with immunotherapy.^4^
